# Relationship Between Aspartate Aminotransferase-to-Platelet Ratio Index and Carotid Intima-Media Thickness in Obese Adolescents with Non-Alcoholic Fatty Liver Disease

**DOI:** 10.4274/Jcrpe.891

**Published:** 2013-09-18

**Authors:** Ahmet Sert, Özgür Pirgon, Ebru Aypar, Hakan Yılmaz, Bumin Dündar

**Affiliations:** 1 Konya Training and Research Hospital, Department of Pediatric Cardiology, Konya, Turkey; 2 Süleyman Demirel University, Faculty of Medicine, Department of Pediatric Endocrinology and Diabetes, Isparta, Turkey; 3 Konya Training and Research Hospital, Department of Radiology, Konya, Turkey; 4 Katip Çelebi University, Faculty of Medicine, Department of Pediatric Endocrinology and Diabetes, İzmir, Turkey

**Keywords:** adolescent, aspartate aminotransferase-to-platelet ratio index, carotid intima-media thickness, insulin resistance, Non-alcoholic fatty liver disease, obesity

## Abstract

**Objective:** There is increasing evidence for an association between non-alcoholic fatty liver disease (NAFLD) and an increased risk of cardiovascular morbidity and mortality. The aim of this study was to investigate the association between aspartate aminotransferase-to-platelet ratio index (APRI) and carotid intima-media thickness (IMT) in obese adolescents with NAFLD.

**Methods:** Seventy-six obese adolescents and 36 lean subjects were enrolled in this cross-sectional single-centre study. The obese subjects were divided into two subgroups based on the presence or absence of fatty liver with high transaminase levels (NAFLD group and non-NAFLD group). Fasting blood samples were assayed for transaminase, glucose, and insulin levels. Insulin resistance was calculated by the homeostasis model assessment (HOMA-IR).

**Results:** APRI values were higher in both obese groups (NAFLD and non-NAFLD) in comparison with the lean group. The NAFLD group had significantly higher APRI values than the non-NAFLD obese group and the lean group. Carotid IMT was higher in both obese groups (NAFLD and non-NAFLD) in comparison with the lean group. The NAFLD group had significantly higher measurements of carotid IMT than the non-NAFLD group and the lean group. APRI was positively correlated with most of the metabolic parameters (total cholesterol, low-density lipoprotein cholesterol, glucose, insulin, HOMA-IR) and with carotid IMT in the NAFLD obese group.

**Conclusions:** This study demonstrated that a significant relationship exists between APRI and carotid IMT in obese adolescents with NAFLD. We suggest that an increased APRI score in obese adolescents with NAFLD can possibly serve to predict a more adverse cardiovascular risk profile.

**Conflict of interest:**None declared.

## INTRODUCTION

Non-alcoholic fatty liver disease (NAFLD) is most commonly associated with obesity, insulin resistance, and the metabolic syndrome. There is increasing evidence for an association between NAFLD and an increased risk of cardiovascular morbidity and mortality. The relationship between NAFLD and cardiovascular risk factors can largely explain the higher risk of cardiovascular disease among patients with NAFLD (1). The measurement of carotid artery intima-media thickness (IMT) is increasingly used to evaluate the cardiovascular risk and target organ damage in adolescents with metabolic abnormalities (2). Recent case-control studies and cross-sectional studies have reported the increased risk of carotid atherosclerosis in NAFLD (3). There are only a few studies that evaluate the association between NAFLD and carotid IMT in children and adolescents using ultrasonography and elevated liver enzymes to diagnose NAFLD (4,5).

It appears that no single clinical or laboratory parameter can reflect the presence of hepatic fibrosis or the severity of fibrosis in children with NAFLD (6). Several studies have suggested that the aspartate aminotransferase-to-platelet ratio index (APRI) may be a useful noninvasive marker of hepatic fibrosis in patients with chronic liver disease (7,8,9,10). However, regarding APRI, only one study has been reported in children with NAFLD. Yang et al (11). showed that, among noninvasive scores, APRI exhibited statistically significant differences between patients with mild fibrosis and those with significant fibrosis. However, the relationship between APRI score and a more adverse cardiovascular risk profile in obese adolescents with NAFLD has not been investigated. In this study, we aimed to determine whether an association exists between APRI and carotid IMT in obese adolescents with NAFLD.

## METHODS

Seventy-six obese adolescents (37 girls and 39 boys) with a mean age of 13.0±1.3 (age range: 12-17) years and a mean body mass index (BMI) of 30.02±3.69 were randomly recruited from obese children who were admitted to our Pediatric Endocrinology Units from November 2011 to April 2012. The obese group was divided into two subgroups: NAFLD patients (16 girls and 25 boys, mean age: 13.1±1.1 years, mean BMI: 30.8±3.9, BMI standard deviation score (SDS): 2.13±0.3) with high alanine aminotransferase (ALT) levels (ALT>40U/L) and ultrasound evidence of fatty changes in the liver, and non-NAFLD patients (21 girls and 14 boys, mean age: 13.1±1.2 years, mean BMI: 29.03±3.19, BMI SDS: 2.08±0.6) with low ALT levels (ALT<40U/L) and without any ultrasound evidence of fatty changes in the liver. The healthy lean adolescents to serve as controls were required to meet the following inclusion criteria: (1) age, 12-17 years; (2) a BMI smaller than the 84th percentile for age and gender based on reference curves for Turkish adolescents; (3) absence of a prior major illness, including type 1 or 2 diabetes mellitus (T1DM or T2DM); (4) low ALT levels (ALT<40U/L) and no ultrasound evidence of fatty changes in the liver. These adolescents (16 girls and 20 boys, mean age: 13.6±1.4 years, mean BMI: 19.50±2.23, BMI SDS: 0.5±0.7) were enrolled for the study from among non-obese healthy adolescents who attended the hospital for minor illnesses such as a common cold, conjunctivitis, or similar mild afflictions. Patients who had any systemic disease, including T1DM or T2DM, those taking medications, or who had a condition known to affect insulin action or insulin secretion (e.g. glucocorticoid therapy, hypothyroidism, Cushing’s disease) were excluded.

The study protocol was approved by the institutional review board of Konya Research Hospital Ethics Committee. Signed informed consent forms were obtained from the parents of the children.

Anthropometric measurements were performed in all subjects. Height was measured to the nearest 0.5 cm on a standard height board. Weight was determined to the nearest 0.1 kg on a standard physician’s beam scale with the subject dressed only in light underwear without shoes. BMI was calculated as weight (in kilograms) divided by height (in meters) squared. Patients with a BMI of ≥95th percentile according to reference curves for Turkish adolescents were accepted as obese (12). Pubertal development stage was assessed by a single pediatric endocrinologist using the Tanner criteria. Staging for sexual maturation was >2 in all patients (Tanner stages II-IV).

After the child had rested for at least 5 minutes and was in a sitting position, diastolic and systolic pressure (mmHg) measurements were taken, using a mercury-gravity manometer and a cuff appropriate for body size.

**Laboratory Assessment**

Fasting blood samples (at 8:00 AM) were obtained in the morning by venipuncture after an overnight fast (at least 12 h). Glucose was determined by the glucose oxidase method. Serum concentrations of total cholesterol, high-density lipoprotein cholesterol (HDL-cholesterol), and triglycerides were measured using routine enzymatic methods with Abbott diagnostics C16000 chemistry analyzer, (USA). Low-density lipoprotein cholesterol (LDL-cholesterol) level was calculated using Friedwald equation. Serum insulin levels were measured by an Immulite immunoassay (Diagnostic Products, Los Angeles, CA, USA). Standard liver function tests (ALT, aspartate aminotransferase (AST), were measured on the same day with an autoanalyzer, and the AST/ALT ratio was calculated. APRI was calculated by the following equation: (AST level/AST upper level of normal/platelet count) × 100 (7).

**Insulin Sensitivity Measurement**

Insulin resistance was estimated using the homeostasis model assessment (HOMA-IR): fasting insulin concentration (µU/mL) x fasting glucose concentration (mmol/L)/22.5 (13). Insulin resistance was defined as a HOMA-IR level greater than 3.16 (14).

**Liver Ultrasonography**

All patients with abnormally high transaminases and abnormal liver ultrasound results were screened for other parameters used in the assessment of liver functions (hepatitis B surface antigen, hepatitis C antibody, prothrombin time, iron, total iron-binding capacity, ferritin, and antinuclear antibodies) with negative results in all subjects. Liver ultrasonography was performed by a trained operator who was blinded to all clinical and laboratory characteristics of the participants. Scans were performed in all subjects using a GE Healthcare Logic 7 (MI, USA) machine, equipped with 7.5 MHz probes in younger children and 5 MHz probes in large-sized or markedly obese adolescents. The presence of NAFLD was assessed by the scoring system defined by Hamaguchi et al (15) according to the hyperechogenicity of liver tissue, and discrepancy between liver and diaphragm and visibility of vascular structures. Scores ranged from 0 to 6 points, and a diagnosis of NAFLD was made if the ultrasonography score was ≥1. The radiologist performing the ultrasonography recorded a sagittal sonogram which showed the liver edge in the right subcostal scan and transverse scan. On the basis of the images, four sonographic findings including hepatorenal echo contrast, bright liver, deep attenuation, and vessel blurring were evaluated. The diagnosis of hepatorenal contrast was based on evident sonographic contrast between the hepatic and right renal parenchyma of the right intercostals sonogram in the midaxillary line. The diagnosis of bright liver was based on abnormally intense, high level echoes arising from the hepatic parenchyma and was graded on a three-grade scale as none, mild or severe, evaluated according to intensity. The diagnosis of deep attenuation was based on evident attenuation of echo penetration into the deep portion of the liver and impaired visualization of the diaphragm. The diagnosis of vessel blurring was based on impaired visualization of the borders of the intrahepatic vessels and narrowing of their lumen. When the hepatorenal echo contrast and bright liver were ≥1, we summed up all scores. When the hepatorenal echo contrast and bright liver were negative, the total score was accepted to be zero. Although liver ultrasonography cannot estimate either fibrosis or inflammation, it has a sensitivity of 89% and a specificity of 93% for detecting histological steatosis. Therefore, the diagnosis of NAFLD is usually made from mild elevations in liver enzymes during a routine blood testing and liver ultrasonography in an overweight or obese child. Elevated ALT was defined as >40 IU/L. All obese patients with NAFLD in our study had high ALT levels.

**Carotid Intima-Media Thickness Measurements**

Carotid ultrasound studies were performed by a single radiologist, who was blinded to the clinical and laboratory status of the patients, using high-resolution B-mode ultrasonography (GE Healthcare Logiq 7, USA) with a high-resolution linear array vascular transducer (14 MHz). An optimal two-dimensional image of the common carotid artery was obtained where both the near and far wall intima/media complex were well visualized. After a 10-min rest and following standard guidelines, the M-mode curser was placed 1 cm proximal to the beginning of the carotid artery bulb during end diastole. Carotid IMT was calculated by taking the mean value of three measurements.

**Statistical Analysis**

Mean and standard deviations (SD) were used as descriptive statistics. Differences in the means of variables were tested using both parametric and nonparametric statistical analyses. SPSS for Windows, version 15 (SPSS, Chicago, IL, USA) was used, depending on the distribution of the variables. The correlations among numerical data were analyzed by the Pearson’s correlation coefficient (r). Multivariate stepwise regression analysis was performed to assess independent predictors of levels of APRI. A p-value of less than 0.05 was considered statistically significant.

## RESULTS

The characteristics of the study population are shown in Table 1. Gender, age, and BMI were similar between the non-NAFLD and NAFLD groups. The control (lean) group included 36 sex-, age- and pubertal stage-matched non-obese healthy subjects without liver steatosis.

The NAFLD obese group had also significantly higher AST, ALT, total cholesterol, LDL-cholesterol, fasting insulin levels than both the non-NAFLD and the lean groups. AST/ALT ratio values were lower in the NAFLD obese group when compared to the non-NAFLD and the lean groups.

**Figure 1:**
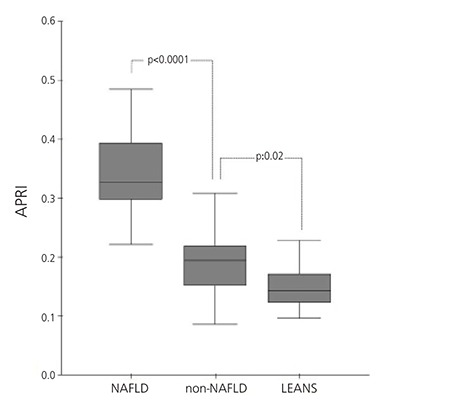
Aspartate aminotransferase-to-platelet ratio index (APRI) in non-alcoholic fatty liver disease (NAFLD), non-NAFLD and lean groups

The lean group had lower HOMA-IR values than both the non-NAFLD and the NAFLD obese groups (0.73±0.29 vs. 3.73±2.95 vs. 5.50±3.35). Moreover, the NAFLD obese group had significantly higher APRI (0.342±0.073 vs. 0.200±0.075 vs. 0.149±0.038) and carotid IMT (0.458±0.043 vs. 0.377±0.017 vs. 0.359±0.012 mm) values than the non-NAFLD and the lean groups (Table 1) (Figure 1).

APRI was positively correlated with most of the cardiovascular risk parameters [total cholesterol (r=0.534,p<0.0001), LDL-cholesterol (r=0.595, p<0.0001), glucose(r=0.656, p<0.0001), insulin (r=0.523, p<0.0001), HOMA-IR(r=0.549, p<0.0001)] and carotid IMT (r=0.917, p<0.0001) in the NAFLD obese group. APRI was positively correlated with total cholesterol (r=0.382, p=0.024) and LDL-cholesterol(r=0.463, p=0.005) also in the non-NAFLD obese group. APRI was not related to any cardiovascular risk parameters in the control group (Table 2).

In the multivariate stepwise regression analysis; APRI score (β=0.917, p<0.001) was positively correlated with carotid IMT in the NAFLD group even after adjusting for age, sex, BMI, systolic and diastolic blood pressures, total cholesterol, triglycerides, LDL-cholesterol and HDL-cholesterol, fasting glucose and insulin, HOMA-IR as co-factors with the total variance explained being 84.1%. In the non-NAFLD group, APRI score (β=0.463, p=0.005) was positively correlated with LDL-cholesterol with the total variance explained being 21.4%.

## DISCUSSION

In this cross-sectional study, we demonstrated a positive significant association between APRI score and carotid IMT in obese adolescents with NAFLD, a finding which suggested that an increased APRI score demonstrated a more adverse cardiovascular risk profile. We think that insulin resistance may have an impact on both APRI and carotid IMT. In our study, a multivariate regression analysis showed that APRI was positively correlated with carotid IMT in adolescents with NAFLD. It is important to remember that we chose for this study young asymptomatic patients who were otherwise healthy apart from having either elevated liver enzymes or a fatty liver on ultrasound.

Non-invasive diagnosis of liver fibrosis has been extensively evaluated in the adult population with NAFLD (16). The use of APRI as a correlate of disease progression is based on findings of liver pathobiology. AST levels rise with the progression of liver disease as a likely result of direct hepatocellular damage and membrane leak although they may normalize in patients with compensated cirrhosis. Platelet counts decrease with progression of liver disease and the resultant changes in splenic blood flow (17). These tests are performed as part of the standard of care for children with liver disease and thus would not result in added cost or effort. The APRI is inherently limited by the use of a standard upper limit of normal value. Past studies have shown that many pediatric patients have normal AST values of 15-20, but an AST of 40 as in our study would likely generate a low APRI value despite the fact that this value would be twice that patient’s normal value (18).Obesity, hypertension, male gender, dyslipidemia, and insulin resistance have been reported to be independent predictors of advanced fibrosis (19). Iacobellis et al (20) identified the independent predictors of hepatic fibrosis in 69 children with non-alcoholic steatohepatitis (NASH) due to NAFLD. In their study, it has been reported that BMI was independently associated with fibrosis.Hypertriglyceridemia is a strong predictor of NAFLD in the general population and in obese children (21,22). Hypertriglyceridemia was also found to be an independent predictor of liver fibrosis in a study of obese adults (23). In our study, APRI was positively correlated with total cholesterol and 

LDL-cholesterol but not with hypertriglyceridemia.

Hyperinsulinemia is a common feature of pediatric NAFLD, and there is some evidence that it may be an independent predictor of liver fibrosis (24). In the present study, APRI was positively correlated with HOMA-IR.

Several mechanisms have been suggested, such as increased oxidative stress and chronic subclinical inflammation as well as decreased concentrations of adiponectin, an adipose-secreted cytokine with antiatherogenic properties (25). Moreover, the presence of an abnormal lipoprotein metabolism or an increase in whole body insulin resistance and/or dyslipidemia could also lead to accelerated atherosclerosis (26). However, these mechanisms are also closely related to other risk factors for atherosclerosis. Therefore, it is unclear whether NAFLD contributes to the development of atherosclerosis directly.

Carotid IMT is a reliable index of subclinical atherosclerosis, and epidemiologic studies have demonstrated that there is a significant association between carotid IMT and cardiovascular disease (27). Francanzani et al (28) reported no difference in carotid IMT levels between patients with simple steatosis and those with NASH, even if these latter patients had higher carotid IMT values than controls. On the other hand, Targher et al (29) reported an independent association of carotid IMT with the liver histology among NAFLD patients. In the present study, we found significant differences regarding carotid IMT levels between obese adolescents with NAFLD and lean groups. In addition, a positive correlation was found between APRI and carotid IMT values in obese adolescents with NAFLD.

Few studies have been published so far on the association between ultrasonographically detected NAFLD and carotid IMT in children (4,5). Pacifico et al (4) showed that obese children with NAFLD had significantly increased carotid IMT than obese children without liver involvement and control children. Similarly, in our study, carotid IMT values were significant higher in both obese groups (NAFLD and non-NAFLD) in comparison with the lean group. Demircioglu et al (5) found an association between fatty liver status and left carotid IMT measured at sites of the common carotid artery, carotid bulb and internal carotid artery in 80 obese children and 30 normal-weight controls. In their study, obese children with NAFLD had higher values of insulin and adiponectin as compared to obese children without NAFLD and controls. In our study, right carotid IMT was significantly higher in adolescents with NAFLD than in those without NAFLD.

Impaired insulin action in the liver results in decreased glucose uptake, decreased oxidative and non-oxidative glucose metabolism (30). These defects of hepatic glucose uptake and metabolism contribute to the development of liver disease (31). With respect to visceral adiposity, there is a great deal of evidence suggesting two strong links with insulin resistance. First, unlike subcutaneous adipose tissue, visceral adipose cells produce significant amounts of proinflammatory cytokines such as tumor necrosis factor-alpha, and interleukins 1 and 6. In numerous experimental models, these proinflammatory cytokines disrupt normal insulin action in fat and muscle cells, and may be a major factor in causing the whole-body insulin resistance observed in patients with visceral adiposity. Secondly, visceral adiposity is related to an accumulation of fat in the liver, a condition known as NAFLD. The result of NAFLD is an excessive release of free fatty acids into the bloodstream (due to increased lipolysis), and an increase in hepatic glucose production, both of which have the effect of exacerbating peripheral insulin resistance and increasing the likelihood of T2DM (32). In NAFLD, hepatic insulin resistance mediates the failure of the insulin-signaling pathway, leading to molecular and cellular changes that result in excess accumulation of triglycerides in the hepatocytes (33). Insulin resistance also stimulates the pro¬duction of free fatty acids and increases lipid deposition in the liver, which, in turn, enhances oxidative stress and consequently results in the development of liver fibrosis. Moreover, insulin resistance is also involved in the proliferation of hepatic stellate cells, which are the main site of deposition of abnormal extracellular matrix in hepatic fibrosis (34). In this present study, we found a correlation between APRI, which is a useful noninvasive marker of hepatic fibrosis and carotid IMT values in obese adolescents with NAFLD. Insulin resistance, hepatic fibrosis and the associated cascade of proinflammatory and profibrogenic pathways generated in the liver might promote increased carotid IMT in NAFLD, although this hypothesis needs validation in larger prospective studies also measuring serum levels and liver expression of proinflammatory and profibrogenic cytokines.

The most important limiting factor of our study was that the stage of NAFLD was not determined by liver biopsy in our patients. Liver biopsy remains the gold standard for characterizing liver histology in patients with NAFLD. However, it is expensive procedure and carries some morbidity and very rare mortality risk, especially in young children. For these reasons, surrogate markers such as ALT/AST ratio or ultrasonography are usually used to detect NAFLD (35). Ultrasonography is a valuable noninvasive method of screening for liver disease. ALT is a widely accepted biomarker for liver fat accumulation; however, it is neither as specific nor as sensitive as biopsy. Measurement of liver enzymes alone is not sufficient for reliable fatty liver screening in overweight children. The findings of Fishbein et al (36) show that enzymatic abnormalities in fatty liver occur only in the severe cases. Mofrad et al (37) report that an entire spectrum of NAFLD can be seen in subjects with normal ALT values.

APRI score is a new marker for prediction of cardiovascular mortality and of hepatic mortality (38). However, a fairly consistent finding relating to the APRI score has been its lack of sensitivity for mild to moderate fibrosis. This finding is consistent with the pathobiology involved, since the increase in AST levels and the fall in platelet counts also occur relatively late in disease progression. In patients with compensated cirrhosis with normal AST values, the APRI score would also be a poor predictor of significant fibrosis. Inflammatory activity also needs to be considered in predicting the progression of the cirrhosis (39).

In conclusion, we found a significant relationship between APRI score and carotid IMT in obese adolescents with NAFLD. However, prospective studies addressing larger groups of patients are necessary to better understand this association.

**Statement of Authorship**

AS and OP carried out the literature review, sample size, data analysis, study design, and drafted the manuscript. EA, HY and BD helped with the design of the study, data analysis and drafting of the manuscript. All other authors participated in study design, completion of the study and finalizing the manuscript. All authors read and approved the final manuscript.

## Figures and Tables

**Table 1:**
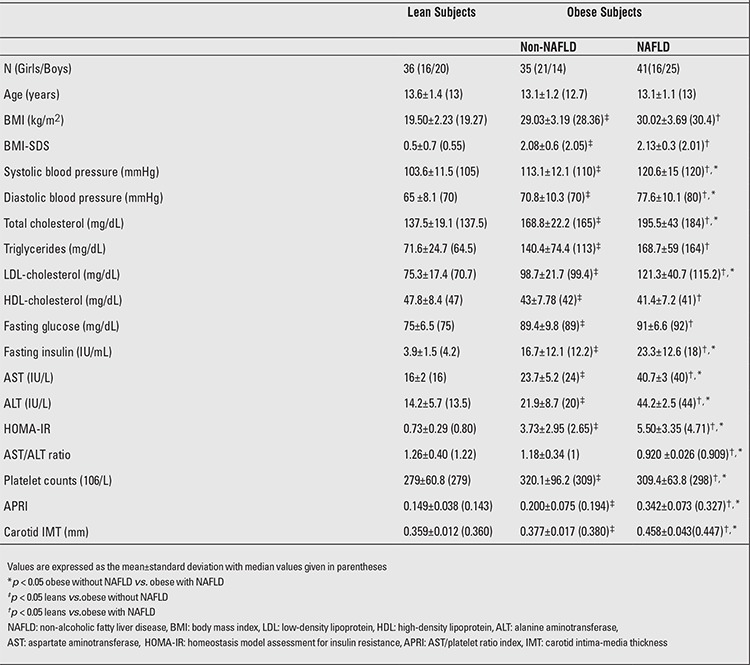
Characteristics of lean and obese adolescents

**Tablo 2:**
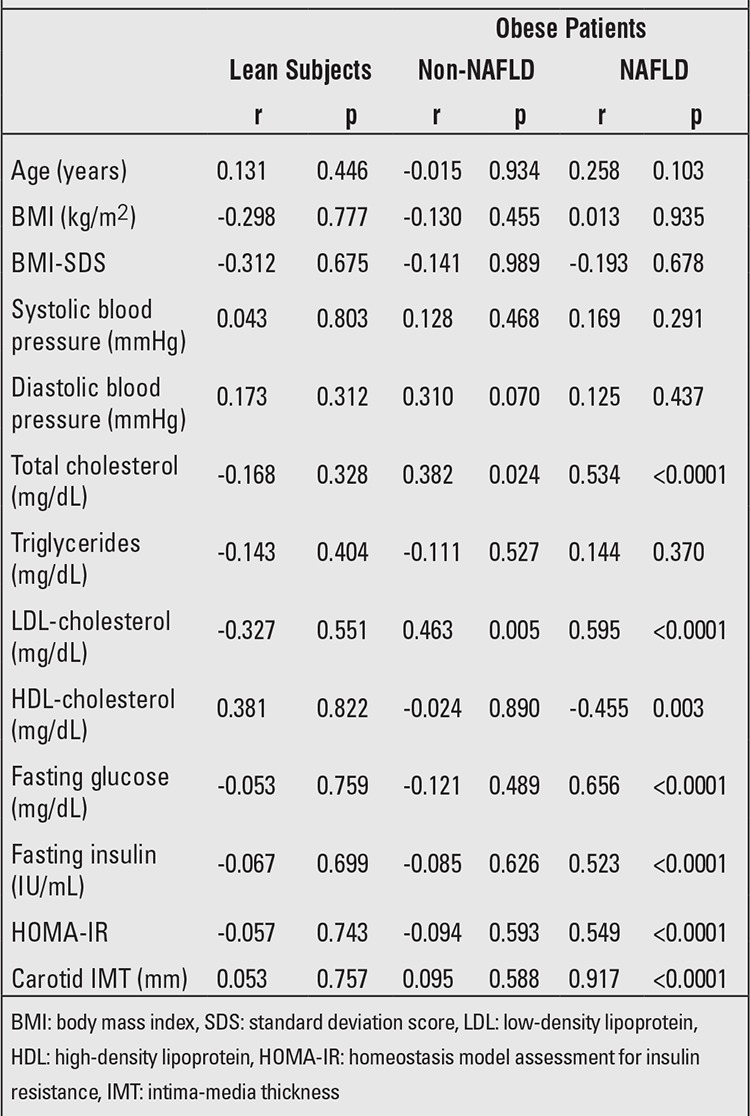
Pearson’s correlations between aspartate aminotransferase-to-platelet ratio index (APRI) and cardiovascular risk parameters in obese adolescents
